# Composites in Aerospace and Mechanical Engineering

**DOI:** 10.3390/ma16227230

**Published:** 2023-11-19

**Authors:** Stelios K. Georgantzinos, Georgios I. Giannopoulos, Konstantinos Stamoulis, Stylianos Markolefas

**Affiliations:** 1 Laboratory for Advanced Materials, Structures, and Digitalization, Department of Aerospace Science and Technology, National and Kapodistrian University of Athens, 34400 Psachna, Greece; 2 Department of Mechanical Engineering, School of Engineering, University of Peloponnesus, 1 Megalou Alexandrou Street, 26334 Patras, Greece; ggiannopoulos@uop.gr; 3 Faculty of Technology, Amsterdam University of Applied Sciences, 1097 DZ Amsterdam, The Netherlands; k.stamoulis@hva.nl; 4 Department of Aerospace Science and Technology, National and Kapodistrian University of Athens, 34400 Psachna, Greece; stelmarkol@uoa.gr

**Keywords:** composites, composite structures, nanocomposites, characterization, modeling, simulation, multiscale methods, structural integrity, damage assessment, NDT/SHM

## Abstract

An important step towards improving performance while reducing weight and maintenance needs is the integration of composite materials into mechanical and aerospace engineering. This subject explores the many aspects of composite application, from basic material characterization to state-of-the-art advances in manufacturing and design processes. The major goal is to present the most recent developments in composite science and technology while highlighting their critical significance in the industrial sector—most notably in the wind energy, automotive, aerospace, and marine domains. The foundation of this investigation is material characterization, which offers insights into the mechanical, chemical, and physical characteristics that determine composite performance. The papers in this collection discuss the difficulties of gaining an in-depth understanding of composites, which is necessary to maximize their overall performance and design. The collection of articles within this topic addresses the challenges of achieving a profound understanding of composites, which is essential for optimizing design and overall functionality. This includes the application of complicated material modeling together with cutting-edge simulation tools that integrate multiscale methods and multiphysics, the creation of novel characterization techniques, and the integration of nanotechnology and additive manufacturing. This topic offers a detailed overview of the current state and future directions of composite research, covering experimental studies, theoretical evaluations, and numerical simulations. This subject provides a platform for interdisciplinary cooperation and creativity in everything from the processing and testing of innovative composite structures to the inspection and repair procedures. In order to support the development of more effective, durable, and sustainable materials for the mechanical and aerospace engineering industries, we seek to promote a greater understanding of composites.

## 1. Introduction

With its ability to provide a strategic advantage in the design and development of high-performance structures, composite materials have become a fundamental component in the advancement of mechanical and aerospace engineering. These materials are complicated due to their heterogeneous compositions, which necessitates a deep comprehension of their inherent properties [1,2,3,4,5]. In order to predict the behavior of composites in real-world applications, researchers use sophisticated characterization techniques to understand the composite’s response to stress, temperature, and environmental variables [6,7,8,9,10].

Beyond the lab, material optimization is being investigated to satisfy the demanding requirements of aerospace applications, where every gram matters. When maintaining or minimizing the overall weight, engineers work to improve mechanical properties including toughness, fatigue resistance, and thermal stability. Modern manufacturing techniques, such as additive manufacturing, which enables the creation of components with intricate geometries and optimal material distribution, are used to achieve this equilibrium [11,12,13,14,15,16].

There are challenges in integrating composites into the current industrial frameworks. When switching to composites from traditional materials, assembly methods, testing procedures, and maintenance protocols must all be reevaluated. To ensure that composite materials perform reliably in engineering applications, new joining, inspection, and repair methods are being developed to accommodate their unique characteristics [17,18,19,20,21,22]. Addressing the durability and damage tolerance of composites is another critical area of focus. The industry seeks to understand the mechanisms of impact resistance and fatigue in composite structures to develop predictive models for their lifespans and reliability. This is particularly important in the aerospace sector, where material failure is not an option. Advanced non-destructive testing methods and real-time monitoring systems are being explored to detect and mitigate damage before it compromises safety [23,24,25,26].

The environmental impact of composite materials is an issue that spans the entire lifecycle, from raw material extraction to end-of-life disposal or recycling. The development of sustainable composites, which includes the use of bio-based resins and recyclable fibers, is a growing field of research. These materials aim to reduce the environmental footprint of composites while maintaining their high-performance characteristics [27,28,29,30].

Multiscale modeling has become an indispensable tool in the study of composites, bridging the gap between molecular-level interactions and macroscopic properties. This approach allows for the simulation of composite behavior across different scales, providing insights into how changes at the nano- or microscale can influence the overall performance of the material. Multiscale models are particularly useful in predicting the mechanical responses of composites under complex loading conditions, informing the design process, and enabling the optimization of material properties [31,32,33,34].

In the broader context, the development of composites is not just a technical endeavor, but also a strategic one. The ability to tailor materials to specific applications opens up new possibilities in design and functionality. As the field of composites continues to mature, it promises to redefine the boundaries of what is achievable in aerospace and mechanical engineering, driving innovation and performance to new heights. The articles on this topic delve into the synergistic effects of these technologies on composite performance, offering insights into how they can be harnessed to meet the specific demands of aerospace and mechanical engineering.

## 2. An Overview of Published Articles

Contribution 1 is encapsulated in the article from the journal *Aerospace*. This research addresses the critical issue of damage tolerance in aeronautical composite structures, particularly focusing on the behavior of stiffened carbon fiber reinforced polymer (CFRP) panels under compression after impact. The study aims to understand the residual strength of these panels when they have sustained damage, which is essential for compliance with damage tolerance requirements in the aerospace industry. The paper details the development of a stiffened specimen for the VERTEX multiaxial test rig, which is a step towards evaluating the damage tolerance of stiffened structures under combined loadings. The specimen design was informed by virtual testing to ensure it would exhibit the desired damage phenomena, specifically the debonding of the stiffener from the center as the first mode of failure. The research involved manufacturing three samples and subjecting them to low-velocity impacts at various locations and energies, followed by compression testing to the point of stiffener debonding under a post-buckling regime of the skin. The results of the tests, including force fluxes and global strains, were obtained from in situ stereo-correlation, showing that different impacts resulted in different types of damage but similar residual strength. This work contributes to the broader understanding of the damage tolerance behavior of stiffened composite structures and their post-buckling capabilities, potentially allowing for more efficient sizing and mass reduction in composite panels.

Contribution 2 is encapsulated in an article from the journal *Applied Sciences*. This work investigates the aerodynamic and structural design of fairings for vehicles, employing the Rankine half-body theory for aerodynamic design—a theory commonly used for aircraft gas turbine engine inlets and turboprop nose cones. The structural design leverages glass fabric composite materials, analyzed under aerodynamic design loads to ensure structural safety. The research culminates in the manufacturing of the fairing using the resin transfer molding method, with the final design being reviewed for its compatibility with existing vehicles and confirmed for safety. The study contributes significantly to the field by applying aircraft drag reduction techniques to commercial vehicles, aiming to improve fuel efficiency and reduce environmental impact. It also addresses the gap in research regarding the overall structural safety and lightening of fairings compared to existing structures.

Contribution 3 is encapsulated in the article from the journal *Materials*. This research delves into the dynamic behavior of sandwich beams that incorporate PMI foam cores when they are subjected to low-velocity impact loading. The study is particularly focused on understanding how these beams sustain and transfer loads upon impact, which is crucial for applications in aerospace, marine, and other engineering fields where such materials are often used due to their advantageous mechanical and physical properties. The paper outlines the experimental procedures used to assess the performance of the sandwich beams under both quasi-static and dynamic loading conditions. Different types of PMI foam cores were tested to analyze the influence of core density and face sheet thickness on the load-sustaining and transfer mechanisms. The experiments involved clamping the beams and subjecting them to impacts at varying velocities to measure the impact force using an accelerometer. The results demonstrated that the impact force and duration increased with the thickness of the face sheet and the density of the core. Moreover, the study observed a transition in failure modes from shear to tensile, attributed to the strength ratio between the bottom face sheet and the core. The findings of this research contribute to a better understanding of the dynamic deformation mechanisms of sandwich beams with PMI foam cores, which is essential for designing more resilient structures in various engineering applications.

Contribution 4 is detailed in the article from the journal J. Compos. Sci. This study explores the blast mitigation performance of sandwich panels by examining various design parameters and core geometries. The research compares three core designs—honeycomb, mushroom, and tubular—to assess their effectiveness in resisting blast loads. The study also considers the impact of plate thickness and the spacing between core structures on the panels’ performance. Using finite element analysis, twenty-seven numerical experiments were conducted. The results were then evaluated through regression analysis. The findings indicate that the tubular sandwich panel exhibited the least deformation and damage while contributing to the highest kinetic energy dissipation. Conversely, honeycomb core structures recorded the highest internal energy dissipation, but also the most deformation and damage. Despite these differences, the study found that core shape and spacing were less influential in resisting blast loads compared to plate thickness, which emerged as the most significant factor.

Contribution 5 is presented in the article published in the journal *Materials*. The study focuses on the optimization of heat treatment conditions for accurately measuring the resin content in CFRP and glass-fiber-reinforced plastic (GFRP) used in construction. The method of thermal gravimetric analysis (TGA) was utilized to determine the optimal temperature and time for heat treatment that would remove the resin without causing loss to the fibers. The research involved incrementally increasing the temperature by 100 °C up to 800 °C and maintaining a heat treatment time of 4 h to observe the degree of thermal decomposition of the resin. It was found that most resin decomposition in CFRP occurred at 300 °C, and at a pyrolysis temperature of 400 °C, the resin was effectively removed with no significant change in weight, indicating no fiber damage. However, at temperatures of 500 °C or higher, partial thermal decomposition of the fibers was observed. This study is significant as it provides a method for determining the resin content in FRP construction materials without compromising the integrity of the reinforcing fibers. The optimized heat treatment conditions can be crucial for ensuring the quality and performance of FRP materials in the construction industry.

Contribution 6 is presented in the article published in the journal *Materials*. This study investigates the thermal buckling behavior of moderately thick laminated conical shells that are reinforced with layers originating from carbon nanotubes (CNTs). This research is significant for applications where these materials are exposed to uniform rises in temperature, such as in aerospace and other high-performance engineering fields. The paper utilizes the Donnell-type shell theory to derive the governing equations for the thermal buckling problem. The Galerkin method is then employed to determine the buckling temperature while considering shear deformation theories (STs). The analysis incorporates different transverse shear stress functions, including parabolic, cosine-hyperbolic, and uniform shear stress functions, to evaluate the buckling behavior. After validating the formulation against the existing literature, the study describes several parametric experiments to explore the effects of CNT patterns, the number of layers, and their arrangement on the uniform buckling temperature (UBT).

Contribution 7 is presented in the article published in the journal *Materials*. This study examines the impact of interface shear strength (IFSS) on the mechanical performance of carbon fiber-reinforced composites with a polyether ether ketone (PEEK) thermoplastic matrix. The research compares two types of laminates: one made with as-received fiber fabrics and the other with fibers that had undergone thermal treatment to remove the original sizing agent. The IFSS was measured using the push-in test, which showed that fibers treated to remove the sizing agent exhibited a 25% higher critical shear stress. Microscopic inspections revealed that untreated specimens were more prone to debonding, leading to a higher crack density. This was detected by the C-Scan technique and was evident in the response of the laminates under tensile tests at ±45° fiber orientation. The desized specimens demonstrated a 37% increase in maximum stress and a 190% increase in strain at break values, confirming that the original fiber sizing weakened the fiber–matrix interface. The study contributes to the understanding of how the fiber–matrix interface affects the mechanical properties of composite materials, which is crucial for their application in the aerospace and automotive industries.

Contribution 8 is encapsulated in the research article published in the journal *Applied Sciences*. The study focuses on the dynamic buckling behavior of composite laminated structures that are reinforced with a honeycomb pattern and filled with viscoelastic material. This research is particularly relevant for engineering applications where such structures are subjected to varying dynamic loads, such as in aerospace, automotive, and civil engineering. The paper presents a mathematical model of the honeycomb-reinforced composite laminated plate, considering the coupling of in-plane forces. The critical buckling load is determined using large deflection theory, and the effective dynamic stiffness modulus of the composite sandwich is obtained through homogenous asymptotic theory. The study also explores the influence of geometric and external load factors on the dynamic buckling load of these composite structures. The numerical analysis conducted in the study provides insights into the parameters that significantly affect the critical buckling loads, such as the axis load parameter, the geometry parameter, the thickness ratio of the core layer, the honeycomb reinforcement’s width, and the dynamic impulse load’s frequency. The findings contribute to the design and optimization of composite structures for enhanced stability under dynamic loading conditions.

Contribution 9 is encapsulated in the research article published in the journal *Materials*. The study addresses the complex mechanical behavior of solid propellants, which are composite materials known for their nonlinear viscoelastic characteristics. This behavior is largely attributed to the cumulative damage process caused by the formation and growth of microflaws within the material. The research presents a series of standard relaxation and uniaxial tension tests on hydroxyl-terminated polybutadiene (HTPB) propellant at different velocities, utilizing digital image correlation (DIC) techniques to capture the deformation. The findings confirmed that the mechanical behavior of the propellant is rate-dependent, with both yield stress and failure stress significantly influenced by tensile velocity. To accurately describe this behavior, the study developed a nonlinear viscoelastic constitutive model that incorporated rate-dependent cumulative damage. This model employed a Prony series representation of viscoelastic material functions and introduced a rate-dependent damage variable to account for the characteristics of the damage accumulation process. Furthermore, a new normalized failure criterion was derived from the proposed damage model, which remained independent of strain rate after normalization. The model’s accuracy and the failure criterion were validated through finite element analysis, showing high agreement with the experimental data under various strain rates. This research contributes to the field by providing a more precise description of the mechanical characteristics of solid propellants, which is crucial for the design and analysis of solid rocket motors.

Contribution 10 is presented in the research article published in the journal *Aerospace*. This study delves into the thermal and mechanical properties of carbon/carbon (C/C) composite materials, which are extensively utilized in aerospace structures that operate in high-temperature environments due to their superior performance characteristics. The paper focuses on the yarn architecture of C/C composites, where fiber bundles cross in various directions, and these are categorized into different types such as 3-D orthogonal, 4-D in-plane, and 4-D diagonal, based on the arrangement of the fiber bundles. The thermo-mechanical properties of these materials can be tailored by manipulating the yarn architecture, the constituent materials (fiber and matrix), and manufacturing parameters like yarn size, yarn spacing, and fiber volume fraction. In this research, geometric models for repeating unit cells (RUCs) are defined according to the yarn architecture of the C/C composite material. The study employs the iso-strain assumption and stress averaging technique to predict the effective stiffness of these materials. Furthermore, it compares and evaluates the thermo-mechanical characteristics of the RUCs based on yarn architecture and fiber volume fraction. The study’s approach allows for the prediction of thermo-mechanical properties of C/C composite materials, which can significantly reduce testing costs and optimize structural weight while ensuring performance. This is particularly beneficial in the preliminary design stages of aerospace structures, where material selection and parameter optimization are crucial.

Contribution 11 is detailed in the article published in the journal *Materials*. This study proposes a micromechanical simulation approach within a multi-scale modeling (MSM) framework that incorporates manufacturing defects, specifically focusing on the effects of void shape on the macromechanical properties of materials. The research includes a case study on a cross-ply laminate, emphasizing the significance of accurate micromechanical geometry and void characteristics for predicting material behavior. A representative volume element (RVE) model was developed using actual micromechanical geometry obtained from micrographs. Voids were introduced into the RVE model based on statistical experimental data, and their impact on fiber distribution and effective macromechanical properties was assessed. The findings suggest that the local void fraction, void size, and void shape significantly influence the effective micromechanical properties. The study underscores the importance of considering these factors in relation to the overall void fraction of an RVE and the actual laminate. The proposed MSM framework demonstrates a robust prediction capability for macromechanical properties and shows promise for industrial application, particularly in identifying weak spots and critical areas in laminate structures on a macro-level.

Contribution 12 is outlined in the article published in the journal *Applied Sciences*. The research focuses on developing a methodology to assess the residual strength of C/SiC ceramic matrix composite panels when exposed to simultaneous thermal and acoustic stresses. This is particularly relevant for aerospace applications where such materials are used in structures that encounter extreme temperature and noise conditions, such as the thermal protection systems (TPS) of hypersonic vehicles. The study uses a 2D plain-woven C/SiC ceramic matrix composite panel as the test subject, applying spatially uniform thermal loading and band-limited Gaussian white noise to evaluate its geometric nonlinear response through numerical simulation. The material properties, including static strength, residual strength, and fatigue life, are characterized under tensile and compression loads at elevated temperatures. A computer code is developed to simulate the fatigue behavior of the composite panels, and the methodology’s accuracy is validated against experimental results from residual strength tests on the panels under the combined loadings. The findings show a good correlation between the predicted residual strength and the experimental data, indicating the reliability of the proposed methodology in assessing the durability of C/SiC composites under harsh operating conditions.

Contribution 13 is presented in the article published in the journal *Materials*. The research investigates the bond–slip behavior at the interface between CFRP and steel, which is a critical factor in the performance of CFRP-strengthened steel structures. The study conducted a series of double-strap experiments on CFRP-strengthened steel plates and employs finite element analysis to simulate the bond characteristics. The experiments revealed that the maximum shear stress at the bonding interface was higher for the Q345B steel specimen compared to the X100 specimen. It was also found that both the initial slip and maximum slip at the interface increased with the thickness of the bonding layer. The finite element model incorporated a maximum stress criterion to simulate the onset of material damage when the nominal stress reached the maximum nominal stress threshold. The model’s effectiveness was validated through a verification test, which confirmed that the modified equations derived from the study aligned well with both the numerical and experimental results. This research contributes to the understanding of the bond performance at the CFRP–steel interface and provides modified equations that can predict the bond–slip properties effectively, which is crucial for the design and assessment of CFRP-strengthened steel structures.

Contribution 14 is encapsulated in the article published in the journal *Materials*. The study explores the challenges of machining CFRPs, which are widely used in various industries due to their superior mechanical properties, but present difficulties due to their anisotropy and inhomogeneity. The research focuses on the slot milling of plain-woven CFRP using polycrystalline diamond (PCD) tools, examining the influence of cutting parameters and tool rake angle on the cutting force and surface roughness. The findings indicate that a PCD tool with a 4° rake angle resulted in a smaller cutting force compared to a 0° rake angle tool, although the rake angle did not significantly affect the surface roughness. The concept of an equivalent cutting area was introduced to analyze the variation in cutting force and surface roughness, revealing that both increase with a larger equivalent cutting area and decrease with a smaller one. The study also discusses the different material removal mechanisms under varying equivalent cutting areas and explains the causes of delamination observed on the top layer after milling.

Contribution 15 is encapsulated in the article featured in the journal *Materials*. The study delves into the integration of shape memory alloys (SMA) with polymer matrix composites (PMC) for use in active aerodynamic systems in the automotive sector. A significant challenge in this integration is the limited strength at the metal–polymer interface, which is crucial for the functionality of SMA-actuated systems. The research focuses on selecting materials with appropriate thermo-mechanical properties to prevent premature activation of the SMA during the polymer setting and to avoid damage to the polymer during the SMA’s thermal activation. Nonstandard samples consisting of SMA wires embedded in cylindrical resin blocks were created for both static and fatigue pullout tests under thermo-mechanical loading. The study also included fully coupled thermo-mechanical simulations with a special constitutive model for SMAs to analyze stress and temperature distribution. The results demonstrate the substantial impact of SMA thermal activation on adhesion strength due to significant recovery forces and temperature increases at the interface. Under static mechanical load, the samples showed a nominal pullout stress of around 940 MPa, which was reduced to 280 MPa under simultaneous thermal and mechanical loads. Additionally, a fatigue run-out of 5000 cycles was achieved at a nominal stress of around 200 MPa when combining thermal activation and mechanical loads. These findings highlight the design constraints of SMA/PMC systems, particularly regarding the maximum allowable stresses during static and cyclic actuation.

Contribution 16 is detailed in the article published in the journal *Applied Sciences*. The study investigates the integration of plasma combustion technology into micro gas turbine engines (GTEs) using biodiesel fuel derived from animal fat, with a focus on enhancing performance, reducing fuel consumption, and minimizing greenhouse gas (GHG) emissions. The research was conducted at Kuwait’s Public Authority for Applied Education and Training, where laboratory design, fabrication, assembly, testing, and evaluation of the results took place. The study found that the use of biodiesel blended fuels resulted in the lowest emissions of sulfur, nitrogen oxides (NOx), and carbon monoxide (CO). The introduction of hydrogen plasma into the biodiesel improved the thermal efficiency, leading to an increase in the compressor inlet and outlet firing temperatures by 13.3 °C and 6.1 °C, respectively. This enhancement in combustion efficiency due to plasma technology also resulted in a thrust increment of 0.2 kgf for the highest loading condition, positively affecting horsepower and overall GTE engine efficiency while also reducing fuel consumption costs.

Contribution 17 is presented in the article published in the journal *Materials*. This study addresses the challenge of fabricating carbon-fiber-reinforced silicon carbide (Cf/C-SiC) composites, which are highly sought after for high-temperature load-bearing applications due to their low density, high strength, and excellent thermal–physical properties. Traditional methods for preparing Cf/C-SiC composites often require high sintering temperatures and pressures, which can limit their practical applications. The research introduced a novel binary binder composed of coal pitch and polysilylacetylene, which served as a carbon source, SiC precursor, and semi-ceramic SiC filler. This innovative approach allowed for the successful incorporation of the SiC phase into C/C composites through a slurry impregnation–hot pressing sintering method. The resulting Cf/C-SiC composites exhibited impressive mechanical properties, with a density of 1.53 g/cm³ and a bending strength of 339 ± 21 MPa. Furthermore, the study investigated the impact of the binary binder on the microstructure, density, and mechanical properties of the composites, providing a promising and efficient method for producing Cf/C-SiC composites with enhanced performance characteristics.

Contribution 18 is encapsulated in the article published in the journal *Materials*. This paper presents the innovative development of hybrid composites with significantly improved interfacial properties, achieved by integrating aligned zinc oxide (ZnO) nanowires with continuous carbon fibers. The atomic layer deposition method was utilized to synthesize nanoscale ZnO seeds uniformly on carbon fibers, followed by the growth of vertically aligned ZnO nanowires using a low-temperature hydrothermal method. The morphology and chemical compositions of the ZnO nanowires were meticulously characterized to assess the quality within the hybrid fiber-reinforced composites. Remarkably, single-fiber fragmentation tests revealed an impressive 286% improvement in the IFSS of the epoxy composites. To further understand the interfacial behavior, a multiscale modeling framework was developed, incorporating a cohesive zone model (CZM) to simulate the interface between the fiber and matrix and an ABAQUS user subroutine for the damage behavior of the fiber. The combined experimental and analytical results underscore the efficacy of the aligned ZnO nanowires in enhancing the key mechanical properties of hybrid fiber-reinforced composites, marking a significant advancement in the field of composite materials.

Contribution 19 is encapsulated in the research article published in the journal *Materials*. The study delves into the thermal decomposition mechanisms of DFTNAN, a novel fluorinated low-melting-point explosive, by comparing it with TNAN under various heating conditions. The research utilized DSC-TG-FTIR-MS and T-jump-PyGC-MS coupling analyses to monitor the thermal decomposition processes and initial reactions. The findings indicate that the presence of fluorine reduces the thermal stability of the molecular structure, shifting the trigger bond from the ortho-nitro group of the ether to the para-nitro group. Upon initial bond breakage, DFTNAN tends to undergo rupture of the dissociative nitro group, leading to significant heat release and subsequent ring opening of the benzene structure. The study also observed major side reactions, including the formation of polycyclic compounds and the migration of fluorine atoms. The research highlights that fluorine not only affects thermal stability, but also alters the reaction pathway, with fluorinated products appearing as fluorocarbons due to the robustness of the C-F bond.

Contribution 20 is detailed in the article published in the journal *Materials*. This research investigates the magneto-thermoelastic interactions within an unbounded medium that encompasses a spherical cavity. The study employed an advanced multi-time-derivative dual-phase-lag thermoelasticity model to analyze these interactions. The spherical cavity’s surface was assumed to be traction-free and was subjected to both constant heating and an external magnetic field. The authors developed a generalized magneto-thermoelastic coupled solution using Laplace’s transform. The graphical representation of the field variables was used to elucidate the effects of the magnetic field, phase-lags, and other parameters on the field quantities. The study validated the present theory by comparing it with the existing literature, demonstrating its relevance and accuracy.

Contribution 21 is encapsulated in the research article published in the journal *Aerospace*. This study focused on the impact of lightning strikes on the structural integrity of honeycomb sandwich composite structures, which are extensively used in aircraft due to their unique performance characteristics. The research aimed to assess the residual mechanical properties of these structures following a lightning strike. The methodology included simulated lightning strike tests on honeycomb sandwich panels, both with and without a carbon nanotube film (CNTF), to evaluate different damage scenarios and the protective effect of CNTF. Subsequently, the residual compressive strength of the damaged panels was predicted using a progressive damage analysis method. The predictions were then validated against experimental results, demonstrating a correlation between the numerical predictions and the actual outcomes. The study found that the size and extent of lightning damage significantly influence the compression damage mode of honeycomb sandwich panels with closed edges.

Contribution 22 is presented in the article published in the journal *Applied Sciences*. The study introduced a novel approach to fabricating load-bearing structures with high mass-specific mechanical performance, suitable for various applications in engineering, architecture, automotive, or aerospace industries. The research focused on additive manufacturing processes, specifically coreless filament winding with fiber composites and laser powder bed fusion with metals, to produce lightweight structures. These processes exhibit unique characteristics that must be considered to successfully integrate multiple materials and methods. The hybrid design approach aims to combine the benefits of different materials to achieve mass savings in load-bearing structures with high mass-specific stiffness, strict geometrical tolerance, and machinability. A digital tool for coreless filament winding was developed to capture the process-specific characteristics and support all project phases. The study demonstrated this by stiffening an aluminum base plate with a coreless wound fiber-composite structure, which was attached using additively manufactured metallic winding pins. The research also introduced the concept of multi-stage winding to minimize fiber–fiber interaction, which can be challenging to predict and control. The results showed a significant increase in component stiffness by a factor of 2.5 with only one-fifth of the mass compared to a state-of-the-art reference. This indicates that the hybrid design approach has substantial potential to enhance performance when process-specific features, interfaces, material interaction, and process interdependencies are considered during the digital design phase.

Contribution 23 is encapsulated in the research article published in the *Materials* journal. This study delved into the hot corrosion characteristics of cobalt-based DZ40M and nickel-based K452 superalloys when exposed to NaCl molten salt at 900 °C, a scenario that simulated the harsh conditions faced by aero-engine turbine blades in marine environments. The research outlines the experimental setup where the superalloys were coated with NaCl salt and subjected to high-temperature conditions to observe the corrosion behavior. The findings reveal that the K452 superalloy, with its higher content of aluminum and titanium, forms a relatively continuous Al_2_O_3_ and TiO_2_ layer that exhibits lower solubility and less damage in Na_2_O, resulting in a lower hot corrosion rate compared to DZ40M. The latter contains higher amounts of carbon, chromium, and tungsten, which influence its corrosion behavior differently. The paper proposes a corrosion mechanism induced by NaCl by comparing the oxidation and hot corrosion behavior of the two alloys, providing insights into their failure processes. This research is significant for the aerospace industry as it offers a theoretical reference for the selection of materials for guide vanes in turbine blades, which are critical for the performance and longevity of aero-engines.

Contribution 24 is detailed in the article published in the *Materials* journal. This study addresses the challenges of machining CFRP, which are widely used in advanced manufacturing fields such as aviation and aerospace due to their favorable properties. The research introduces a novel gradual-removal reverse edge milling cutter designed to improve the hole-making quality in CFRP. The study established finite element method models to simulate the reverse helical milling process with both the new cutter and a conventional flat-bottomed reverse edge milling cutter under ultrasonic vibration. Comparative cutting experiments were conducted to assess the performance of both cutters. The findings indicate that the new milling cutter can effectively transfer part of the cutting task from the peripheral edge to the end edge, reducing wear on the peripheral edges and improving the quality of the hole at the outlet. This is attributed to the cutter’s design, which allows the peripheral edge to be dominated by shear failure, leading to a significant enhancement in the outlet quality.

Contribution 25 is encapsulated in the research article published in the *Aerospace* journal. This study investigates the nonlinear behaviors and interactions among the constituents of composite material structures under tensile load, focusing on fiber-reinforced composite laminates with central holes. The research employer a multiscale damage model using the generalized method of cells (GMC) and a lamina-level progressive damage model, both based on the thermodynamic Schapery Theory (ST) at either the micro-level or the lamina level. The study’s approach involved predicting the load versus displacement curves and failure modes of the open-hole laminates by using two progressive failure models. These models were compared with the Hashin–Rotem progressive failure model and experimental results. The findings demonstrate that the ST-based method can capture the nonlinear progressive damage evolution states and failure states of the composite at both the lamina level and the multiscale level. The paper also presents damage contours and failure paths, contributing to our understanding of the mechanical response and failure mechanisms of composite structures.

Contribution 26 is presented in the article published in the *Materials* journal. This research addresses the challenge of enhancing the strength and ductility of magnesium (Mg) matrix composites by incorporating CNTs as a reinforcing agent. The study explores the use of electrophoretic deposition (EPD) to achieve a uniform dispersion of CNTs within a metal matrix, followed by spark plasma sintering (SPS) to synthesize the layered CNTs/Mg composites. The article details the methodology, including the preparation of CNTs, the EPD process for layering, and the subsequent SPS. The results demonstrate that the composite samples sintered at 590 °C exhibited improved strength and ductility compared to pure Mg and those sintered at 600 °C. Additionally, the composites rolled by 40% showed significantly higher strength without a substantial decrease in ductility. The study also examined the damping properties of the composites, revealing that the damping-test-temperature curve increases with temperature and that the composites maintain excellent damping properties at room temperature. The research concludes that the layered structure of the CNTs/Mg composites, prepared via EPD, can effectively disperse CNTs and enhance the material’s mechanical properties while preserving ductility, offering potential applications in aerospace and other advanced manufacturing fields.

Contribution 27 is highlighted in the article published in the *Materials* journal. The study delves into the damping characteristics of CFRP raft frames, which are pivotal in vibration isolation in mechanical systems. Utilizing composite laminated plate theory and a strain energy model, the research examined how different layups affect the damping capacity of the raft frame and its components, such as the top/bottom plate and I-support. The study employed finite element analysis (FEA) and a damping ratio prediction model to compare the theoretical results with the experimental data. It was found that the layup of CFRP laminates significantly influenced the damping ratio of the raft frame, with the maximum error in the first-order natural frequency and damping ratio for the top/bottom plate being 5.6% and 15.1%, respectively. The I-support showed a 7.5% maximum error in the first-order natural frequency between FEA and test results, indicating a larger error in the damping ratio due to stress concentration. The research concluded that the damping performance of the raft frame is affected by the arrangement of the I-support, and the simulation analysis aligned well with the experimental outcomes. This study provides valuable insights for enhancing the damping performance of CFRP raft frames, which could have significant implications for their application in various manufacturing sectors, including aerospace.

## 3. Conclusions

The series of studies presented under this topic offer a comprehensive examination of the advancements in composite materials and their applications in aerospace and mechanical engineering. The research contributions collectively address the critical aspects of material characterization, damage assessment, and structural integrity, providing insights into the multifaceted nature of composite materials.

The collective findings from these studies underscore the importance of innovative design, precise material characterization, and advanced manufacturing techniques in the development of composite materials for aerospace applications. The integration of experimental, numerical, and theoretical approaches across these contributions provides a holistic view of the current state and future directions of composite material research in the aerospace sector.
